# Bulk pharmaceutical research data management

**DOI:** 10.1155/S1463924682000406

**Published:** 1982

**Authors:** Hugh B. Woodruff, Patricia C. Tway, George V. Downing, Jack P. Gilbert

**Affiliations:** Merck Sharp & Dohme Research Laboratories PO Box 2000 Rahway New Jersey 07065 USA


Journal of Automatic Chemistry, Volume 4, Number 4 (October–December 1982), pages 161–164

### Bulk pharmaceutical research data management

**Hugh B. Woodruff, Patricia C. Tway, George V. Downing and Jack P. Gilbert**

*Merck, Sharp & Dohme Research Laboratories, PO Box 2000, Rahway, New Jersey 07065, USA*

The sophistication of analytical instrumentation and the ability to generate data with these instruments have increased significantly in recent years [1 and 2]. These new instruments permit a huge amount of data to be generated in a very short period of time, yet in many cases no computerized system has been developed or implemented for automatic storage and retrieval of the analytical results. Most of the data are still hand-transcribed onto an analytical form and the papers subsequently filed away. Many samples that are submitted to an analytical chemistry or quality-control department require 20 or more different analytical tests performed in a variety of laboratories for complete evaluation. As a result, sample tracking to allow quick determination of the current status often requires excessive amounts of telephoning, paper shuffling, and rummaging through files. The test results for each sample or batch of material are found on a separate piece of paper so that comparison of results between different lots requires one to search through the file to gather all the data, and then to put the results in tabular form. Recent government regulations have greatly increased the responsibilities of laboratory and management personnel to document completely all analytical testing results, both sample results and instrument calibrations. Optimum use of analytical results and of manpower can be achieved with a computerized data storage and retrieval system.

Reduced costs of computer hardware and increased proficiency of data management software have enabled a number of laboratories to streamline their operations and improve efficiency [3–8]. Packaged data management software designed for laboratory use is commercially available (for example, LIMS from Perkin-Elmer Corporation and LABMAN from Spectrogram Corporation) and software designed to aid the user in building his or her own data-base is also commercially available (for example, SIMILE from Za-Tro Corporation and RS/1 from Bolt Beranek and Newman Inc.).

Whether one purchases commercially available software or produces customized programs to meet specific needs, the human engineering aspects of the final product introduced into the laboratory and the office should be the principal considerations [9–11]. Human engineering, human interface, and user friendly are commonly used terms or 'buzzwords'. However, too often these terms are not put into practice. This paper describes a system implemented in the Analytical Research Department at Merck, Sharp & Dohme Research Laboratories, with the major emphasis of the presentation focused on human rather than software considerations.

### Description of the programs

One step in a departmental goal of distributed, compatible automation was the development of adequate data management programs. The initial objective of this work was to produce tabulations of analytical results from Merck's pilot plant control laboratory samples of final bulk chemicals. Approximately 300 results would be added to the table monthly. Previously, such tabulations were generated manually, which was an extremely tedious and sometimes error-prone operation. Eventually, all analytical results from the pilot plant control laboratory (2100 assays/month) will be stored on the computer.

The use of such a computer program to tabulate analytical results meant that technicians, scientists, and managers would all be required to interact with the computer. The selection of computer and programming language was of little importance to the system's users, as long as their interactions with the computer were easy to learn and friendly. Many early laboratory computer systems were failures because of one fatal shortcoming, namely they required humans to adapt to the computer's needs rather than the programs adapting to the users' needs [11].

Since no readily accessible commercial data management package could adequately meet the needs of the department, a group of programs accessed by a command file called PPCL (standing for pilot plant control laboratory) was written and implemented on a CDC Cyber 173 computer. The programs are coded in BASIC, but do not require the user to know any programming language or to have any prior familiarity with computers.

Interactions with users in all PPCL programs follow a friendly question-and-answer dialogue style. Ziegler's paper [11] on the role of the human interface in laboratory computer systems highlights some rules necessary for a successful question-and-answer dialogue. One important rule is to distinguish between the occasional and the frequent user. Occasional users need reminders on how to use the system via a 'HELP' command or a similar approach; but long and involved explanations will annoy the experienced user, so they should be available as options.

PPCL adheres to this rule through the use of commands and options. The prompt to the user is usually as simple as 'ENTER COMMAND'. Experienced users remember the available commands and options and need no additional prompting. Novices can obtain assistance by entering 'HELP', 'H', or '?'.

Figure 1 shows the process through which information is obtained from the user and the table displayed or changed as requested by the user. To initiate the process, the operator enters the only Cyber command he or she needs to know, 'CALL, PPCL'. Tables of information on a specific compound are accessed via a catalogue of identifiers. Legal identifiers are the compound name or internally assigned designators, an 'MK' number, an 'L' number, or a project number. Allowing the user to access a table by a variety of identifiers is simple to implement—its importance to ease of use and operator acceptance is obvious. Many individuals will use the system and not all of them remember the same designator. Requiring them to use a single type of identifier for a table would have been an example of forcing the human to adapt to the needs of the computer.

All tables are constructed so that the tests performed are the

0142-0453/82/0404 0161 $05·00 © 1982 Taylor & Francis Ltd

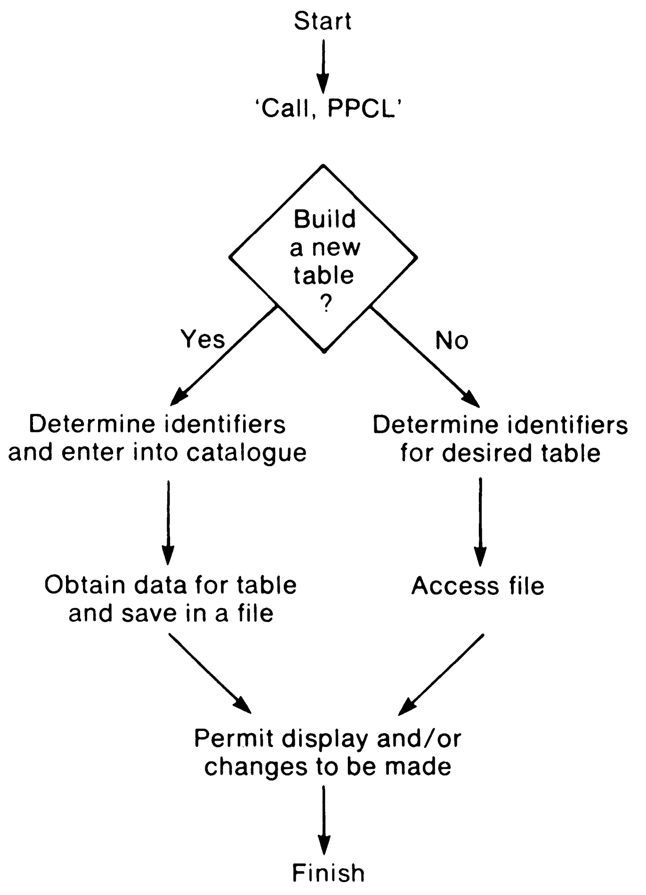

*Figure 1. The way the user obtains information from PPCL.*

row headers, the lot numbers are the column headers, and the body of the table contains assay results or dashes (—) for tests not yet performed. Once acceptable minimum and maximum specifications have been established for a test on the compound in question, these values are added to the table. A maximum of five characters is permitted for each specification and the maximum for other entries is eight characters. Test names and comments longer than eight characters may be entered, but only the first eight characters appear in the table.

The list of commands and options currently available in PPCL is shown in figure 2. To aid in describing figure 2, a

'LIST'

  All

  Row(s)

  Column(s)

  Lots

  Tests

'ADD'
  Row
  Column

'DELETE'
  Row
  Column

'FIND'

'CHANGE'

'UPDATE'

'MOVE'
  Row
  Column

'QUIT'

*Figure 2. The commands and options currently available in PPCL.*

portion of a table for MK0421 is shown here as table 1. Except for 'QUIT', all commands require additional information before executing; for example, once the proper table has been accessed, to produce the print-out shown in table 1, the user might pursue the following dialogue:

ENTER COMMAND (L,A,D,F,C,U,M,Q)? LIST
LIST OPTIONS ARE:
ALL, TESTS (OR SPECS), LOTS (OR BATCHES), ROW #, OR COLUMN #
ENTER LIST OPTION (A,T,L,R#, C#)? COLUMN
UP TO 5 COLUMNS MAY BE LISTED SIMULTANEOUSLY.
ENTER 1 COLUMN NUMBER PER LINE ("0" WHEN THROUGH).
ENTRY #1? 43
ENTRY #2? 44
ENTRY #3? 45
ENTRY #4? 46
ENTRY #5? 47
COMMAND ACCEPTED: LIST COLUMN(S) 43 44 45 46 47
(User responses are indicated by underlining.)

The occasional user is led through the dialogue and does not need to memorize things—the computer issues necessary prompts. However, the experienced user would be annoyed by the excessive printing and waiting between replies. For the experienced user, PPCL allows commands and options to be entered concurrently and with a minimum of restrictions. To do so, the command must precede the option(s) and they must be separated by one or more blanks. An added simplification is that only the first letter of any command or option is required; hence 'LIST', 'L', or 'LXYZ' all result in the 'LIST' command being implemented.

To produce table 1, the experienced user might type:

LIST COLUMN 43; 44; 45; 46; 47

or

L C 43; 44; 45; 46; 47

or

L C 43–47.

PPCL attempts to adapt to the needs of the human rather than the reverse.

Entering a 'LIST LOTS' (or 'L L') command produces a listing of rows 1–4 for all sample lots and includes the column number, lot number, pilot plant control laboratory number, pilot plant batch number, and the entire comment. The result of a 'LIST TESTS' (or 'L T') command is shown in table 2. In this case, both the abbreviated and complete test names appear. Using the 'LIST' command coupled with the appropriate options, the operator can display the results in virtually any form desired.

The remaining commands (except for 'QUIT') allow the user to edit the table as required. 'ADD' is used when the analytical tests are added to the compound specifications ('ROW' option) or when samples from a new lot are submitted and assayed ('COLUMN' option). After an 'ADD COLUMN' (or 'A C') command, the program displays the new column number and asks for the lot number, pilot plant control laboratory number, pilot plant batch number, and a comment. The program then asks for the test results for each analytical test in the compound specifications. For example:

```
ENTER COMMAND (L,A,D,F,C,U,M,Q)? A C
COMMAND ACCEPTED: ADD COLUMN
COLUMN 47
LOT NUMBER? 123
PPCL NUMBER? 8102367
PP BATCH NUMBER? 23
COMMENT? EXAMPLE
APPEARANCE RESULT? CFM
ASSAY BY HCLO4 TITRATION DRY BASIS
RESULT? 100·2
HPLC WEIGHT % ASSAY VS L...22 RESULT? 99·6
    ⋮
```

'DELETE' allows the user to eliminate a complete row or column of data from a table following extra questioning as a safeguard against mistaken deletions. The command is used to remove the results of analytical tests which are no longer used to monitor sample purity.

MOVE permits rearrangements of the row and column ordering for cosmetic or substantive reasons. For example, all the results on a particular sample lot (for example, stability studies) can be put in adjacent columns or all the liquid chromatographic assay results for pure compound and for different impurities can be placed in adjacent rows.

Probably the two most commonly used editing commands are 'FIND' and 'UPDATE' because the most frequent function performed by the operator is adding a new test result to the table. Using table 1 as an example, if a loss on drying (LOD) result has just been obtained for lot 14, one could enter the value using the 'UPDATE' command:

UPDATE ROW 13 COLUMN 45 (or U R 13 C 45).

At this point the user would be prompted to add the value to the table.

If, however, a listing such as shown in table 1 were not readily available, one would not be willing to wait patiently while it was printed to determine the correct row and column numbers for the 'UPDATE' command. 'FIND' obviates this problem by allowing the user to tell the program to search the table for the appropriate character strings. 'FIND LOD' and 'FIND 14' would quickly inform the user that row 13 and column 45 were the correct values.

Similarly, a manager might be asked to determine the current status of lot 14. By using the 'FIND' command followed by a 'LIST COLUMN 45' command, this task would be accomplished quickly.

### The importance of human engineering

One final illustration of adapting software to meet the needs of the user involves the 'CHANGE' command. Initially a comprehensive, row-oriented 'CHANGE' command similar to or superior to the one found on many computer editors was available in PPCL. 'CHANGE 25/XYZ/ABC/ALL' would

*Table 1. Assay results for certain lots of compound MK-0421.*

COMMAND ACCEPTED: LIST COLUMN(S) 44 45 46 47 48
MK0421 L15473901D 27200421&& ENALAPRIL MALEATE

| | | | 44 | 45 | 46 | 47 | 48 |
|---|---|---|---|---|---|---|---|
| 1 LOT # | MIN | MAX | 17 | 14 | 34 | 35 | 36 |
| 2 PPCL # | SPEC | SPEC | 8100376 | 8100377 | 8111175 | 8111248 | 8111588 |
| 3 PP BATCH | ------- | ------- | IYR STAB | IYR STAB | 14-1 | 15-IETAD | 16-IETOA |
| 4 COMMENT | 8 CHARS→ | | 20 NOV 81 | 20 NOV 81 | 15 DEC 81 | 17 DEC 81 | 4 JAN 82 |
| 5 APPEAR | CFM | CFM | CFM | CFM | CFM | CFM | CFM |
| 6 HCLO4TIT | 99·0 | 101·0 | 100·0 | 100·5 | 100·5 | 100·7 | 100·3 |
| 7 LCWT.%22 | 98·0 | 101·5 | 99·4 | 98·3 | 99·7 | 98·7 | 100·2 |
| 8 LC-421 | 98·0 | 100·0 | 99·3 | 99·3 | 99·5 | 98·5 | 99·0 |
| 9 PHENESTE | 0 | 1·0 | — | 0·68 | ND | 0·34 | 0·0 |
| 10 LCKETO | 0·0 | 0·5 | — | ND | ND | 0·16 | 0·0 |
| 11 LCMK0422 | 0 | 2·0 | ND | — | ND | 0·07 | 0·05 |
| 12 LC RSS | 0 | 1·0 | 0·7 | ND | ND | 0·8 | 0·86 |
| 13 LOD | 0 | 0·5 | — | — | 0·14 | 0·68 | 0·29 |
| 14 ROI | 0 | 0·5 | — | — | 0·03 | 0·21 | 0·09 |
| 15 METALS | 0 | 10·0 | — | — | < 10·0 | < 10·0 | < 10·0 |
| 16 SPECROT | −41·0 | −43·5 | −42·6 | −42·1 | −41·9 | −43·4 | −42·9 |
| 17 IR | CFM | CFM | — | — | CFM | CFM | CFM |
| 18 TLC | SS | 1·0 | SS | SS | SS | 2TR | SS |
| 19 KF | 0 | 0·5 | — | — | 0·1 | 0·18 | 0·68 |
| 20 NEPHELOS | 0 | 25·0 | — | — | 20·0 | 18·0 | 20·0 |
| 21 COLOR | 0 | 50·0 | — | — | 0 | 4·0 | 12·0 |
| 22 PH | 2·4 | 2·8 | — | — | 2·5 | 2·6 | 2·6 |
| 23 NICKEL | 0 | 20·0 | — | — | < 10·0 | ND | < 20·0 |
| 24 CH3CN | 0 | 0·10 | — | — | 0·02 | NIL | NIL |
| 25 HEXANE | 0 | 0·10 | — | — | 0·003 | 0·01 | NIL |
| 26 MEOH | 0 | 0·10 | — | — | 0·001 | 0·43 | 0·11 |
| 27 NBUTANOL | 0 | 0·10 | — | — | — | 0·13 | 0·015 |
| 28 DISC | 0 | < 1·0 | — | — | 1 | < 1·0 | < 1·0 |
| 29 X-RAY | CFM | CFM | — | — | SIMILAR | SIMILAR | SIMILAR |
| 30 INIT MP | 141·0 | 144·0 | — | — | 141·5 | 141·5 | 141 |
| 31 FINAL MP | 143·0 | 146·0 | — | — | 143·0 | 142·5 | 141·5 |
| 32 PSASLOPE | 0 | 2·0 | — | — | 0·3+/−0·5 | PENDING | 0·7+/−0·3 |
| 33 PSAEXT | INFO | INFO | — | — | 2·6+/−0·1 | PENDING | 2·6+/−0·1 |
| 34 NAOHTIT | 99·0 | 101·0 | 100·1 | 100·1 | 100·9 | 100·9 | 100·8 |

*Table 2. Test names and specifications for compound MK-0421.*

COMMAND ACCEPTED: LIST TESTS

MK0421 L15473901D 27200421&& ENALAPRIL MALEATE

| ROW | NAME | MIN | MAX | 'FULL TEST NAME |
|---|---|---|---|---|
| 5 | APPEAR | CFM | CFM | APPEARANCE |
| 6 | HCLO4TIT | 99·0 | 101·0 | ASSAY BY HCLO4 TITRATION DRY BASIS |
| 7 | LCWT.%22 | 98·0 | 101·5 | HPLC WEIGHT % ASSAY VS L...22 |
| 8 | LC-421 | 98·0 | 100 | AREA % MK0421 HPLC |
| 9 | PHENESTE | 0 | 1·0 | PHENETHYLESTER AREA % BY HPLC |
| 10 | LCKETO | 0·0 | 0·5 | DIKETOPIPERAZINE AREA % BY HPLC |
| 11 | LCMK0422 | 0 | 2·0 | MK0422 AREA % BY HPLC |
| 12 | LC RSS | 0 | 1·0 | RSS ISOMER AREA % ISOMER BY HPLC |
| 13 | LOD | 0 | 0·5 | % LOSS ON DRYING 2 HR. 60 DEGREES <5 MM PRESSURE |
| 14 | ROI | 0 | 0·5 | % RESIDUE ON IGNITION (SO4) |
| 15 | METALS | 0 | 10 | PPM HEAVY METALS |
| 16 | SPECROT | −41·0 | −43·5 | DEGREES SPECIFIC ROTATION ALPHA D 25C (C=1 METHANOL) DRY BASIS |
| 17 | IR | CFM | CFM | INFRARED SPECTRUM NUJOL MULL CONFORMS TO REFERENCE |
| 18 | TLC | SS | 1·0 | % IMPURE BY THIN LAYER CHROMATOGRAPHY CH3CL:CH3OH:C2H402 90:10:1 |
| 19 | KF | 0 | 0·5 | % WATER BY KARL FISCHER |
| 20 | NEPHELOS | 0 | 25 | NEPHELOS VALUE OF 1% AQ. SOLUTION |
| 21 | COLOR | 0 | 50 | COLOR A%*10 3RD 440 NM 1% AQ. SOLUTION |
| 22 | PH | 2·4 | 2·8 | PH 1% AQ. SOLUTION |
| 23 | NICKEL | 0 | 20 | PPM NICKEL BY AA OR COLORIMETRIC |
| 24 | CHHCN | 0 | 0·10 | % ACETONITRILE BY GC |
| 25 | HEXANE | 0 | 0·10 | % HEXANE BY GC |
| 26 | MEOH | 0 | 0·10 | % METHANOL BY GC |
| 27 | NBUTANOL | 0 | 0·10 | % N-BUTANOL BY GC |
| 28 | DISC | 0 | <1·0 | EXTRANEOUS MATTER DISC [δ] FOR 2 GRAMS |
| 29 | X-RAY | CFM | CFM | X-RAY PATTERN |
| 30 | INIT MP | 141·0 | 144·0 | INITIAL MELTING POINT 2 DEGREE RANGE |
| 31 | FINAL MP | 143·0 | 146·0 | FINAL MELTING POINT |
| 32 | PSASLOPE | 0 | 2·0 | PHASE SOLUBILITY IN ACETONITRILE:ISOPROPYLACETATE 1:1 % SLOPE |
| 33 | PSAEXT | INFO | INFO | PSA EXTR. SOLUB. IN ACETONITRILE: ISOPROPYLACETATE 1:1 |
| 34 | NAOHTIT | 99·0 | 101·0 | ASSAY BY NAOH TITRATION |

cause all occurrences of 'XYZ' to be converted to 'ABC' in row 25. (Of course, the occasional user could be led through the process by informative dialogue.) At the time when this all-purpose 'CHANGE' command was installed in PPCL, the 'UPDATE' command did not exist since the programmer had not realized a need for it. One could add a new assay result using the 'CHANGE' command, although experience proved that it was difficult to do so.

To update table 1 as described earlier by adding an 'LOD' assay result for lot 14 using the CHANGE command, the dash currently in the table needs to be changed to the assay result. While the 'CHANGE' command allowed such a change to be made, it was necessary for the user to determine which occurrence of '—' in row 13 corresponded to column 45. In other words, the user had to list row 13, count the number of dashes, and enter the appropriate 'CHANGE' command format.

Rather than quietly accepting the inconvenience being forced upon them by the computer, the users complained and suggested an 'UPDATE' command as a superior alternative. Since the human engineering and human interfacing aspects of PPCL have at all times been deemed to be the top priorities, the 'UPDATE' command was added to the programs. 'CHANGE' still exists and is a powerful command, although its only remaining use is to correct errors in abbreviated and full test names.

### Conclusions

The introduction of this data management package to the Analytical Research Department at Merck, Sharp & Dohme Research Laboratories has eliminated much of the hand transcription, paper shuffling, and file searching inherent in the old system. Analytical data are entered as soon as the tests are completed so that it is easy to determine which tests have been completed and their results, and which tests are still pending on a sample. Since the computerized data-base is available on a time-sharing system, it can be accessed by scientists and managers located in different buildings. This is done over 'phone lines using terminals.

Human engineering and the human interface are crucial aspects of any successful computer system. The programs must be designed with the user in mind (human engineering) and the interaction between the user and the computer must be friendly (human interface). Since PPCL places top priority on both items, it has proven to be a convenient data management package that has been accepted well by laboratory personnel.

### References

1. Malmstadt, H. V., *Analyst*, **28** (1980), 1018.
2. Stockwell, P. B., *Journal of Automatic Chemistry*, **1** (1978), 10.
3. Struthers, S., *Industrial Chemical News*, **2** (1981), 1.
4. Fok, J. S. and Abrahamson, E. A., *American Laboratory*, **7** (1975), 63.
5. Dessey, R. E. and Starling, M. K., *Analytical Chemistry*, **51** (1979), 924A.
6. Woodruff, H. B., Caldwell, W. B., Singleton, B., Downing, G. V., Rosenberg, A. S., Baron, S. F. and Ocker, W. A., *Chemical, Biomedical and Environmental Instrumentation*, **10** (1980), 353.
7. Binkley, D. P. and Major, H. W., *American Laboratory*, **13** (1981), 66.
8. Grebel, S. B. and Sharrar, C. E., *American Laboratory*, **13** (1981), 130.
9. Stockwell, P. B., *Journal of Automatic Chemistry*, **1** (1979), 239.
10. Foreman, J. K., *Journal of Automatic Chemistry*, **2** (1980), 11.
11. Ziegler, E., *Analytica Chimica Acta Computer Techniques and Optimization*, **122** (1980), 315.

